# Assessing the efficacy of natural soil biotin on soil quality, microbial diversity, and *Rhododendron simsii* growth for sustainable landscape architecture

**DOI:** 10.3389/fmicb.2024.1421647

**Published:** 2024-08-07

**Authors:** Zhiyan Teng, Lan Chen, Sheng Li, Kexuan Pan, Dandan Liu, Zaiyuan Gu, Yijie Wang, Li Huang, Yunwen Chen

**Affiliations:** ^1^College of Agriculture and Biotechnology, Zhejiang University, Hangzhou, China; ^2^College of Landscape Architecture, Zhejiang A&F University, Hangzhou, China; ^3^Aupro (Hangzhou) Ecological Industry Operations Co., Ltd., Hangzhou, China

**Keywords:** natural soil biotin, sustainable landscape architecture, soil quality improvement, *Rhododendron simsii*, soil microbial diversity, enzyme activity

## Abstract

Fertilization significantly influences soil quality and its sustainable use in urban garden maintenance. The widespread application of inorganic fertilizers has raised ecological concerns due to their potential environmental impacts. Organic fertilizers, while beneficial, often have slow effects and are costly. Biofertilizers, with their eco-friendly nature and low carbon footprint, are gaining attention for their multifaceted role in supporting plant growth. Despite the focus on fruit trees, vegetables, and medicinal plants, ornamental plants have been understudied. This study aims to evaluate the efficacy of a novel microbial fertilizer, ‘natural soil biotin’, on Rhododendron plants, specifically the Azalea hybrid ‘Carnation’. The study employed a comparative approach to assess the impact of different fertilization strategies on soil properties, microbial diversity, enzyme activity, plant morphology, and physiological parameters. The application of ‘natural soil biotin’ was compared with the use of inorganic and organic fertilizers. The combined application of ‘natural soil biotin’ was found to effectively enhance soil properties and mitigate the impact of other fertilizers on soil pH. It also improved the relative abundance of beneficial microbial groups such as Proteobacteria, Ascomycota, and Basidiomycota. Furthermore, the mixed application significantly increased the activities of urease and sucrase in Rhododendron plants, which promoted their growth, development, and stress resistance. The results indicate that the mixed application of ‘natural soil biotin’ with inorganic and organic fertilizers not only improved the soil quality but also enhanced the efficiency of fertilizer utilization. This approach led to increased economic and environmental benefits in Rhododendron cultivation. The findings contribute to the foundation for soil improvement and ecological restoration, suggesting that ‘natural soil biotin’ could be a promising alternative or supplement to traditional fertilization methods in sustainable landscape architecture.

## Graphical abstract

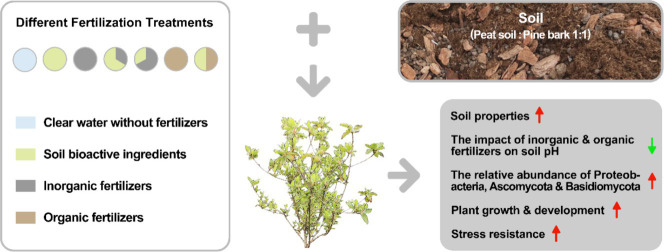

## Highlights

- A novel microbial fertilizer boasts a rich composition of diverse organic matter-degrading enzymes.
- The mixed application of natural soil biotin effectively improved soil properties.
- Natural soil biotin increased the relative abundance of *Proteobacteria*, *Ascomycota*, and *Basidiomycota* in the rhizosphere soil.
- Natural soil biotin improved the growth and ornamental value of *Rhododendron simsii*.

## 1 Introduction

*Rhododendron simsii*, a member of the *Rhododendron* genus in the *Rhododendron* family, is recognized for its evergreen or deciduous shrub-like or small tree-like structure. Renowned for its vibrant flower colors, diverse flower types, and graceful appearance, it has long been favored for landscaping and container gardening, particularly in China, where it is considered among the top 10 traditionally famous flowers (Nie et al., 2024). The extensive germplasm resources of *Rhododendron* have garnered global attention, indicative of its widespread visibility. Beyond its ornamental significance, *Rhododendron* also has notable economic value, driving extensive research across various disciplines, including cultivation, phytochemistry, taxonomy, reproductive and molecular biology, and the microbial ecology of the rhizosphere (Basnett and Ganesan, 2022; Cheng et al., 2024; Duarte et al., 2024). Moreover, *Rhododendron* has a particularly strong demand for fertilization during its peak growth seasons, adhering to the principle of ‘sparse yet frequent application’ to optimize growth and vitality.

Fertilizers serve not only as a nutrient source vital for flower growth but also exert a profound influence on their metabolic processes and ornamental qualities. While conventional chemical inorganic fertilizers offer short-term boosts to agricultural productivity, their inherent limitations cannot be overlooked. These fertilizers pose significant risks of environmental contamination, contributing substantially to carbon emissions. Prolonged reliance on such fertilizers may exacerbate issues like soil compaction and declining soil fertility over time (Gerke, 2022). In comparison, organic fertilizers offer the potential for recycling livestock manure, straw, and other waste materials, thereby enhancing soil conditions. Ideally, they should emerge as the favored option for fertilizer application. However, regulatory oversights in China regarding animal feed have led to the inclusion of excessive heavy metals and antibiotics, resulting in the quality of organic fertilizers being diminished and posing increased risks upon application (He et al., 2023). Moreover, organic fertilizer products are often costly and exhibit slow fertilization efficiency, prompting farmers to favor inorganic alternatives. However, excessive reliance on chemical fertilizers and the application of inadequately fermented or immature organic fertilizers can lead to a sharp elevation in nitrogen (N) or salt levels within the soil, which, in turn, disrupts the soil microbial ecology, rendering the soil infertile and jeopardizes its capacity to sustain plant growth (Li et al., 2022; Zhang C. et al., 2022). Challenges regarding soil management not only impact the green development of the ecological environment but also amplify the financial burden associated with conventional organic and inorganic fertilizer applications, thereby impeding the sustainable advancement of landscape architecture. Consequently, the quest for an economical and environmentally friendly alternative fertilizer solution emerges as a pressing imperative, particularly concerning the cultivation of flowers such as azaleas and the sustainable evolution of agricultural production.

Compared to traditional fertilizers, biofertilizers, characterized by their eco-friendly attributes, offer the potential for low input and high output. They not only yield significant economic benefits but also play a pivotal role in environmental preservation. Aligned with the trend of sustainable development, biofertilizers exhibit vast application prospects. Biofertilizers, as an innovative category of biological fertilizers, leverage the metabolic activities of diverse microorganisms to deliver targeted fertilization benefits in agricultural practices, enhancing crop productivity sustainably (Pu et al., 2022). Studies have shown that even the application of general biofertilizers can suppress soil-borne fungal pathogens, bolster beneficial microbial populations, improve soil structure, and support plant survival and the growth of green crops (Wei et al., 2020; Zhang et al., 2020). Moreover, biofertilizers capitalize on soil organic matter to enrich the soil with secondary organic compounds and increase total N levels, thereby improving soil fertility (Wei et al., 2020). The external application of these biofertilizers has been demonstrated to boost plant yields by stimulating photosynthesis, sugar content, vegetative growth, and advancing the phenological stages, ultimately enhancing the overall quality of the produce (Zhao et al., 2018; Jia et al., 2020). These microorganisms within biofertilizers, each with distinct characteristics, facilitate the transformation of nutrients initially inaccessible to plants in the soil, such as insoluble minerals and organic matter. Through the decomposition activities of these active microorganisms, these nutrients are converted into forms readily absorbed and utilized by plants. Simultaneously, these microorganisms exhibit antagonistic properties against soil-borne pathogens, effectively mitigating the incidence of plant diseases and pests while fostering enhanced nutrient absorption and utilization by plants (Hou et al., 2022; Solanki et al., 2022). This study introduces a groundbreaking microbial fertilizer known as ‘natural soil biotin’ derived from the extraction and cultivation of natural soil. Enriched with a spectrum of organic matter-degrading enzymes, this product rapidly decomposes organic substances, fostering a symbiotic relationship within the ecosystem. ‘Natural soil biotin’ significantly enhances the soil’s microbial and nutritional profile, promoting robust root development in garden plants. Notably, ‘natural soil biotin’, is a lab-processed product that is both environmentally friendly and non-hazardous. It is distinguished by its abundance of oligosaccharide metabolites, a diverse array of microorganisms, trace elements, readily available nutrients, and fermentation by-products and secondary metabolites from its fungal consortia. The formula includes a comprehensive range of microbial communities, such as aerobic and anaerobic bacteria, as well as extremophiles that thrive at both high and low temperatures. Demonstrating exceptional environmental adaptability, ‘natural soil biotin’ retains its efficacy and stability across a wide temperature range from 0°C–70°C and under varying dissolved oxygen levels of 0.2 mg·L^−1^ or higher. This adaptability sets it apart from conventional biofertilizers, ensuring consistent performance in diverse conditions.

Despite the numerous advantages of biofertilizers, certain limitations remain in both research and application. Firstly, further investigation is warranted into the theoretical research on microbial fertilizer mechanisms. While domestic experts and scholars have made notable progress in this domain, additional exploration is necessary to elucidate the alterations in plant root microbial communities’ post-application of biofertilizers and the mechanisms underlying their synergistic interactions with bacterial strains (Kong and Liu, 2022; Wang K. et al., 2023). Secondly, previous studies have predominantly concentrated on enhancing the yield and quality of vegetables while paying less attention to ornamental species (Teng et al., 2024). Consequently, the theoretical framework for microbial fertilizer application in ornamental plants remains relatively underdeveloped. Additionally, there is a pressing need to enhance the stability of microbial fertilizer products and to establish refined quality standards. The key technology of microbial fertilizer involves combining and preserving bacterial strains, which imposes higher requirements on production processes and equipment. To ensure microbial fertilizer quality, it’s necessary to specify quantitative indicators of target bacteria in field applications within product standards (Bebber and Richards, 2022). Moreover, public understanding of microbial fertilizer remains incomplete, potentially creating a gap between research and production. Effective microbial fertilizer application requires consideration of various factors, with selection based on crop and soil characteristics, necessitating region-specific application standards and plans (Hasan et al., 2023).

In this study, the focus was on commonly cultivated garden flowers and trees, specifically *Rhododendrons*, to investigate the effects of a new microbial fertilizer known as ‘natural soil biotin’ on their growth, development, and soil quality. By comparing different fertilization treatments and analyzing their impacts on the physicochemical properties of the cultivation substrate, microbial community diversity, soil enzyme activity, and *Rhododendron* growth and ornamental value, the aim was to identify the optimal fertilization method. Overall, this research contributes to reducing fertilizer usage in landscape architecture, enhancing economic and ecological benefits, and laying a foundation for environmental improvement and ecological restoration.

## 2 Materials and methods

### 2.1 Plant materials

The tested plants comprised one-year-old potted cuttings of *Rhododendron Azalea hybrid* ‘Carnation’, cultivated under uniform conditions. The 2-gallon pot is filled with a balanced potting mixture, crafted from a 1:1 ratio of peat soil and pine bark, and supplemented with approximately 6 kg of stroma per pot. This mixture boasts the following fundamental physicochemical properties: pH 5.78 ± 0.01, organic matter content 401.7 ± 37.6 g·kg^−1^, total N content 9.75 ± 0.14 g·kg^−1^, hydrolyzable N content 328.7 ± 27.0 mg·kg^−1^, available phosphorus (P) content 556.7 ± 18.5 mg·kg^−1^, and available potassium (K) content 212.3 ± 10.3 mg·kg^−1^.

The liquid microbial fertilizer ‘natural soil biotin’, comprising various complex microbial communities, humic acids, and enzymes, was supplied by Hangzhou Aupro Ecological Industry Operations Co., Ltd. The inorganic fertilizer, labeled as NPK compound fertilizer 15:15:15 with total nutrients ≥45%, was procured from Xinyangfeng Agricultural Technology Co., Ltd. Additionally, sheep manure organic fertilizer with an organic matter content of ≥40% was obtained from Ulanqab Lvxiang Agricultural Production Materials Co., Ltd.

### 2.2 Plant growth response to fertilization strategies

A pot experiment was conducted to assess the impact of various fertilization treatments on the growth and quality of *Rhododendron simsii* plants. The following 8 treatments were conducted: (1) Control: watering with clear water only, devoid of fertilizers; (2) T1: applying soil bioactive ingredients alone, comprising base fertilizers at a rate of 50-fold liquid for moistening the substrate, supplemented with monthly topdressing fertilizers at the same rate, amounting to a total of 500 mL of topdressing per pot; (3) T2: applying inorganic fertilizers alone, comprising base fertilizers at a rate of 4 g, with monthly topdressing fertilizers applied at 2 g during the growth period; (4) T3: Mixed application of inorganic fertilizers and soil bioactive ingredients, utilizing base fertilizers at 4 g of inorganic fertilizers mixed with 50-fold liquid of soil bioactive ingredients for substrate moistening, and monthly topdressing fertilizers at 2 g of inorganic fertilizers mixed with 50-fold liquid of soil bioactive ingredients; (5) T4: Application of reduced inorganic fertilizers mixed with soil bioactive ingredients, with base fertilizers at 4 g of compound fertilizers mixed with 50-fold liquid of soil bioactive ingredients for substrate moistening, and monthly topdressing fertilizers at 1 g of compound fertilizers mixed with 50-fold liquid of soil bioactive ingredients; (6) T5: applying organic fertilizers alone, involving base fertilizers at a rate of 120 g, supplemented with monthly topdressing fertilizers at 30 g; (7) T6: Mixed application of organic fertilizers and soil bioactive ingredients, utilizing base fertilizers at 120 g of organic fertilizers mixed with 50-fold liquid of soil bioactive ingredients for substrate moistening, and monthly topdressing fertilizers at 30 g of organic fertilizers mixed with 50-fold liquid of soil bioactive ingredients; (8) T7: Application of reduced organic fertilizers mixed with soil bioactive ingredients, with base fertilizers at 120 g of organic fertilizers mixed with 50-fold liquid of soil bioactive ingredients for substrate moistening, and monthly topdressing fertilizers at 15 g of organic fertilizers mixed with 50-fold liquid of soil bioactive ingredients.

**Figure 1:**
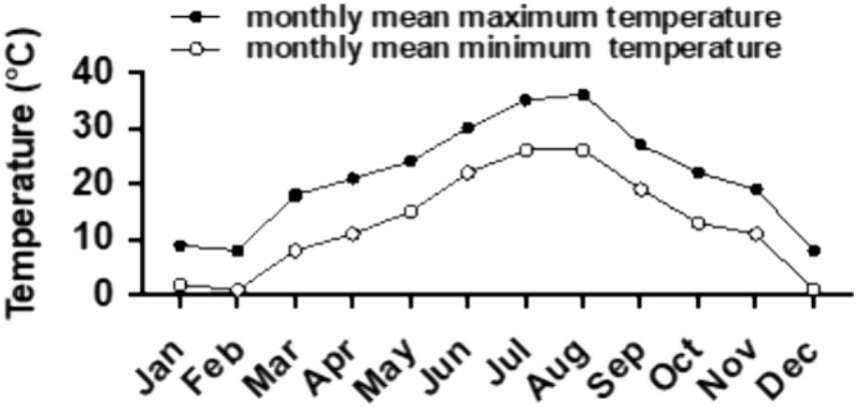
Historical monthly temperature trend of Haining City (originated from the network: https://lishi.tianqi.com/haining/202405.html).

The experiment took place from March 2022 to March 2023 at Shenlongwan Ecological Park in Haining, Jiaxing, Zhejiang Province, situated in the northern Hangjiahu Plain at 30.49059°N, 120.64219°E. The region experiences a subtropical monsoon climate, characterized by an average annual temperature of 15.9°C (Figure 1) and an average annual precipitation of 1,187 mm. The tested plants were placed in an open-air nursery in the park. During periods of intense summer sunlight and high temperatures, black shading nets were used to protect them. Daily standardized cultivation and management practices were implemented throughout the duration of the experiment.

### 2.3 Collection and determination of soil samples for physicochemical indicators

Twenty-four soil samples were systematically gathered from distinct areas—east, south, west, and north fields—of the study site. Each soil sample was taken randomly from five pots per treatment, with the surface layer meticulously cleared away to access the rhizosphere soil. This was extracted using the root shaking technique, homogenized, and any plant debris was meticulously sifted out. The collected samples from these pots were then uniformly mixed and apportioned into three replicates for each treatment. The samples were bifurcated for different analyses: one set was air-dried at room temperature and stored in sealed bags for the assessment of soil physicochemical properties and nutrient levels. The other set was cryopreserved at −80°C in an ultra-low-temperature freezer for future examination of microbial community diversity and soil enzymatic activities. This methodical approach ensured comprehensive data collection for a thorough analysis.

The physicochemical properties of soil samples from each treatment group were assessed following national standard methods. Soil pH was determined using the suspension potential method with a soil-water ratio of 1:2.5 by using a model pHS-25 acidity meter (Shanghai Precise Instruments., China). Organic matter content was determined using the K dichromate oxidation-external heating method (Sun et al., 2018). Total N and hydrolyzable N were determined using the Kjeldahl method (Mühlhauser et al., 1987). Available P was determined using the sodium bicarbonate-molybdate antimony colorimetric method (Zhang et al., 2016), while available K was determined using the ammonium acetate-flame photometric method (Zhou et al., 2021). The parameters including the content of organic matter, total N, hydrolyzable N, available P, and available K were measured by Standard Sci-Tec Innovation (Qingdao) Pharmaceutical Technology Co., Ltd.

### 2.4 High-throughput sequencing analysis of rhizosphere soil microbial community structure and diversity

Soil samples from the rhizosphere of each treatment were collected and forwarded to Beijing Biomarker Technologies Co., Ltd. for sequencing analysis. DNA extraction from the rhizosphere soil of *Rhododendron* plants subjected to different treatments was conducted using the TGuide S96 MagBead Soil Genomic DNA Extraction Kit (Tiangen Biotech (Beijing) Co., Ltd.). Each sample was replicated three times. DNA concentration and purity were assessed using a NanoDrop 2000 UV–Vis Spectrophotometer (Thermo Scientific, Wilmington, United States), and DNA integrity was verified using 1.8% agarose gel electrophoresis.

Primers were designed based on conserved regions, with sequencing adaptors added to their ends. The sequence of bacterial 16S rRNA was specifically amplified using the forward primer 338F (5′-ACTCCTACGGGAGGCAGCA-3′) and the reverse primer 806R (5′-GGACTACHVGGGTWTCTAAT-3′). Additionally, the sequence of the fungal ITS region (internal transcribed spacer) was specifically amplified using the forward primer ITS1F (5′-CTTGGTCATTTAGAG GAAGTAA-3′) and the reverse primer ITS2R (5′-GCTGCGTTCTT CATCGATGC-3′). The PCR system comprised 10 μL total volume, including 5^−^50 ng of DNA, 0.3 μL of each primer (10 μM), 5 μL of KOD FX NeoBuffer, 2 μL of dNTP (2 mM each), 0.2 μL of KOD FX Neo polymerase, and ddH_2_O to make up to 10 μL. The PCR amplification program consisted of pre-denaturation at 95°C for 5 min, denaturation at 95°C for 30 s, annealing at 50°C for 30 s, extension at 72°C for 40 s, with 25 cycles, and final extension at 72°C for 7 min, and preservation at 4°C. Fungal diversity analysis was subsequently conducted on the BMK cloud platform. The raw sequence data was deposited in the National Center for Biotechnology Information (NCBI) Sequence Read Archive (SRA) database.^1^ We have included sample-wise details of all SRA submissions in Supplementary material, including the number of reads generated and the Phred score for each sample, for the convenience of readers as Supplementary Tables 1, 2 and Supplementary Tables 3, 4.

The PCR products were purified, quantified, and standardized to create a sequencing library. This library underwent initial quality control, and only qualified libraries were sequenced using the Illumina NovaSeq 6000 platform. High-throughput sequencing produced raw image data files, which were processed to generate raw sequencing sequences through base calling. Initially, the raw data was filtered using Trimmomatic 0.33 (Bolger et al., 2014), followed by identification and removal of primer sequences using Cutadapt 1.9.1 (Martin, 2011). Subsequently, the dada2 method in QIIME2 2020.6 was employed for noise reduction (Callahan et al., 2016; Bolyen et al., 2019), paired-end sequence assembly, and removal of chimera sequences, resulting in the final set of effective data (Non-chimeric Reads).

The sequences were clustered at a 97% similarity level using USEARCH 10.0 (Edgar, 2013), with a default threshold of 0.005% of the total number of sequences used to filter OTUs (Bokulich et al., 2013). Subsequently, taxonomic annotation of the feature sequences was performed using the Silva.138 database as a reference with a naive Bayes classifier (Edgar, 2013). Alpha diversity and Beta diversity analyses were conducted using the QIIME2 software.^2^

### 2.5 Measurement of soil enzyme activity

In order to evaluate the fertility and overall soil quality of the rhizosphere soil, soil samples from the root zone of each treated *Rhododendron* plant were collected. Superoxide dismutase (SOD) activities were assessed using ultraviolet spectrophotometry (Yu et al., 2023), and urease (UE) activities were determined using indophenol blue colorimetry (Tavares et al., 2021). Additionally, sucrase (SC) activities were measured using 3,5-dinitrosalicylic acid colorimetry (Ge et al., 2018). The parameters including the activity of SOD, UE, and SC were measured by Suzhou Comin Biotechnology Co., Ltd.

### 2.6 Measurement of plant growth indicators

During the cultivation experiment, plant height, crown width, and ground diameter were measured every 2 months, while growth indicators such as the number and length of new branches were assessed at the end of the growth period. Plant height, canopy width, and ground diameter were measured using a steel tape measure (accuracy, 1 mm), a steel tape measure, and an electronic Vernier caliper (accuracy, 0.01 mm), respectively. Other indicators were quantified through visual inspection. During the flowering period of the *Rhododendron*, the number of flowers per inflorescence, flower diameter, and flowering period were observed and recorded. The number of flowers per inflorescence was determined through visual inspection. Flower diameter was measured using an electronic Vernier caliper (accuracy 0.01 mm) at the maximum diameter of the fully open flower. The flowering period was calculated as the duration from 30% budburst to the wilting of most flowers and the loss of ornamental value.

### 2.7 Measurement of physiological indicators on plant leaves

#### 2.7.1 Chlorophyll content

After 1 year of cultivation, leaves from *Rhododendron* plants in each treatment were collected from various directions, including the east field, south field, west field, and north field. The midrib was excised, and the leaves were washed with distilled water before measuring the chlorophyll content. Chlorophyll extraction was performed using the 50% acetone-ethanol mixture grinding method, and the chlorophyll content of the samples was accurately determined using ultraviolet spectrophotometry as described by Ling and Chang-bin (2018). After centrifugation at 4,000 rpm for 10 min at 25°C, 2.0 mL of the supernatant was taken for analysis with an acetone-ethanol mixture as the blank. The Shimadzu UV-1800 spectrophotometer (Shimadzu UV-1800, Tokyo, Japan) measured optical densities at 665, and 649 nm to calculate the concentrations of chlorophyll *a*, *b*, and total chlorophyll according to the formula:

Chlorophyll *a* concentration: C_a_ = 13.95 × A_665_-6.88 × A_649_, unit mg‧L^−1^.
Chlorophyll *b* concentration: C_b_ = 24.96 × A_649_-7.32 × A_665_, unit mg‧L^−1^.
Total chlorophyll concentration: C_t_ = C_a_ + C_b_ = 6.63 × A_665_ + 18.08 × A_649_, unit mg‧L^−1^.
The parameters of chlorophyll content were measured by Suzhou Comin Biotechnology Co., Ltd.

#### 2.7.2 Physiological indicators of cold resistance

At 40 days of age, mature and healthy leaves from various aboveground nodes of each treated plant were harvested and stored at −20°C for subsequent determination of soluble sugar and soluble protein content, the levels of which can reflect plant stress, nutrient status, and overall health.

The soluble sugar content of the samples was assessed using the anthraquinone coloring method. Initially, soluble sugar was extracted from the sample and subsequently measured using a commercial kit (Suzhou Keming Bio-Tech Co., Ltd.). The soluble sugar content was calculated using the following formula: Soluble sugar (mg·g^−1^ fresh weight) = [(ΔA + 0.07) ÷ 4.275 × V1] ÷ (W × V1 ÷ V2) = 2.34 × (ΔA + 0.07) ÷ W. In the equation: V1 represents the volume of sample added, 0.04 mL; V2 represents the volume of extract added, 10 mL; Cpr represents sample protein concentration, mg‧mL^−1^; W represents the fresh weight of the sample, g.

The soluble protein content of the samples was determined using the Coomassie brilliant blue method. Initially, soluble protein was extracted from the sample and subsequently measured using a commercial kit (Suzhou Keming Bio-Tech Co., Ltd.). The soluble protein content was calculated using the following formula: Cpr (mg·g^−1^) = (△A + 0.0007) ÷ 7.1265 × V total ÷ W = 0.1403 × (△A + 0.0007) ÷ W. In the equation, V total is the volume of extract (1 mL), and W represents the mass of the sample (g).

### 2.8 Statistical analysis

Three biological and technical replicates were used for each experiment, with five plants per repetition. Camera pictures were captured using a Canon EOS 600D camera. Graphs were plotted using Excel 2019 (Microsoft, Redmond, WA, United States) and assembled using Microsoft PowerPoint 2019. The data are expressed as means ± SD, computed using SPSS 29.0 analysis software (IBM, Chicago, IL, United States), and subjected to analysis of variance to ascertain statistical significance. Statistical significance was considered at the 95% confidence level (*p* < 0.05). Community analysis plots for each treatment sample were created using the R programming language tool. Adobe Photoshop 2020 was employed to make adjustments and merge the actual photos.

## 3 Results

### 3.1 Effect of different fertilization treatment combinations on rhizosphere soil physical and chemical properties

To assess the impact of natural soil biotin on soil properties and plant growth, soil pH and nutrient content were examined in *Rhododendron* cultivation under various fertilization treatment combinations (Table 1). Soil pH in CK and T1 increased by 0.10 and 0.15, respectively, compared to the initial pH value of 5.78, remaining within the optimal pH range for *Rhododendron* growth. Application of inorganic fertilizers, including T2, T3, and T4 led to a significant decrease in soil pH, whereas application of organic fertilizers, including T5, T6 and T7 resulted in a significant increase in soil pH. In comparison with T2 (received inorganic fertilizer), T3 (received inorganic fertilizer and mixed natural soil biotin) demonstrated an increase by 1.03-fold, while T4 (received reduced inorganic fertilizer and mixed natural soil biotin) had its pH increase by 1.04-fold. Conversely, compared to T5 (received organic fertilizer), T6 (received organic fertilizer and mixed natural soil biotin) exhibited a pH decrease by 1.0-fold, while T7 (received reduced organic fertilizer and mixed natural soil biotin) showed a pH decrease by 0.98-fold. Relative to CK, the application of T3 significantly boosted soil organic matter content, available P and K content. While T3 and T4 did not significantly alter soil alkaline N content compared to T2, T6 and T7 did not significantly alter soil alkaline N content compared to T5, they did significantly reduce total N content.

**Table 1:** Effects of different fertilization treatment combinations on soil nutrient content.

Treatment	pH value	Organic matter	Total N	Alkaline N	Available P	Available K
		(g·kg^−1^)	(g·kg^−1^)	(mg·kg^−1^)	(mg·kg^−1^)	(mg·kg^−1^)
CK	5.88 ± 0.01^d^	416.7 ± 9.2^c^	9.29 ± 0.17^abcd^	641.3 ± 12.5^a^	150.0 ± 4.0^f^	245.7 ± 9.3^e^
T1	5.93 ± 0.02^c^	467.0 ± 13.5^a^	9.32 ± 0.14^abc^	600.3 ± 7.1^a^	99.9 ± 3.2^g^	244.3 ± 9.7^e^
T2	4.94 ± 0.01^g^	424.3 ± 9.5b^c^	9.41 ± 0.02^ab^	644.3 ± 44.5^a^	277.0 ± 12.1^a^	485.7 ± 7.4^a^
T3	5.08 ± 0.02^f^	445.7 ± 23.9^ab^	9.20 ± 0.05^bcd^	607.0 ± 26.5^a^	261.0 ± 13.2^b^	483.0 ± 1.7^a^
T4	5.15 ± 0.01^e^	425.7 ± 10.2^bc^	9.13 ± 0.04^d^	610.3 ± 7.8^a^	246.7 ± 1.5^c^	339.7 ± 3.1^b^
T5	6.32 ± 0.01^a^	422.3 ± 8.7^c^	9.33 ± 0.08^ab^	607.7 ± 4.7^a^	201.0 ± 6.0^d^	337.3 ± 4.0^b^
T6	6.30 ± 0.01^a^	421.0 ± 11.1^c^	9.24 ± 0.11b^cd^	611.3 ± 40.3^a^	175.7 ± 4.0^e^	303.0 ± 7.5^c^
T7	6.18 ± 0.02^b^	429.0 ± 7.8^bc^	9.17 ± 0.03^cd^	604.0 ± 27.8^a^	158.3 ± 7.4^f^	263.3 ± 4.9^d^

T4, Half-supplied inorganic fertilizer + natural soil biotin; T7, half-supplied organic fertilizer + natural soil biotin; T3, inorganic fertilizer + natural soil biotin; T2, inorganic fertilizer; T1, natural soil biotin; CK, no fertilization; N, nitrogen; T6, organic fertilizer + natural soil biotin; T5, organic fertilizer; P, phosphorus; K, potassium. Different lowercase letters indicated a significant difference at 0.05 level.

Fertilizer application significantly influenced the activities of catalase, urease, and sucrase in the soil (Table 2). Organic fertilizers increased catalase and sucrase activities, whereas inorganic fertilizers decreased the activities of catalase, urease, and sucrase. The mixed application of natural soil biotin partially enhanced catalase and sucrase activities. These findings indicate that inorganic fertilizers notably boosted available nutrient content in the soil, while organic and natural soil biotin applications increased soil organic matter and total N content. Moreover, organic fertilizer application enhanced catalase, urease, and sucrase activities, whereas inorganic fertilizer application reduced these activities. However, the mixed application of natural soil biotin improved catalase and sucrase activities.

**Table 2:** Effects of different treatments on enzyme activities in rhizosphere soil of *Rhododendron.*

Treatment	CAT (μmol·d^−1^·g^−1^)	UE (μmol·d^−1^·g^−1^)	SC (mg·d^−1^·g^−1^)
CK	63.04 ± 0.11^b^	316.67 ± 8.73^a^	167.27 ± 6.95^b^
T1	62.42 ± 0.23^c^	291.88 ± 12.27^b^	147.47 ± 7.91^c^
T2	60.91 ± 0.41^d^	79.94 ± 3.18^f^	35.20 ± 2.38^e^
T3	60.79 ± 0.15^d^	140.91 ± 8.98^e^	78.38 ± 5.15^d^
T4	62.50 ± 0.06^c^	165.22 ± 7.92^d^	86.77 ± 6.34^d^
T5	63.18 ± 0.31^ab^	290.78 ± 16.19^b^	217.47 ± 9.59^a^
T6	63.55 ± 0.25^a^	260.61 ± 9.6^c^	210.47 ± 11.81^a^
T7	63.15 ± 0.24^ab^	256.32 ± 9.82^c^	213.89 ± 12.91^a^

CAT, Catalase; T4, half-supplied inorganic fertilizer + natural soil biotin; T7, half-supplied organic fertilizer + natural soil biotin; T3, inorganic fertilizer + natural soil biotin; T2, inorganic fertilizer; T1, natural soil biotin; CK, no fertilization; T6, organic fertilizer + natural soil biotin; T5, organic fertilizer; SC, sucrose; UE, urease. Different lowercase letters indicated a significant difference at 0.05 level.

### 3.2 Effect of different fertilization treatment combinations on the bacterial community in the rhizosphere soil of *Rhododendron*

**Figure 2:**
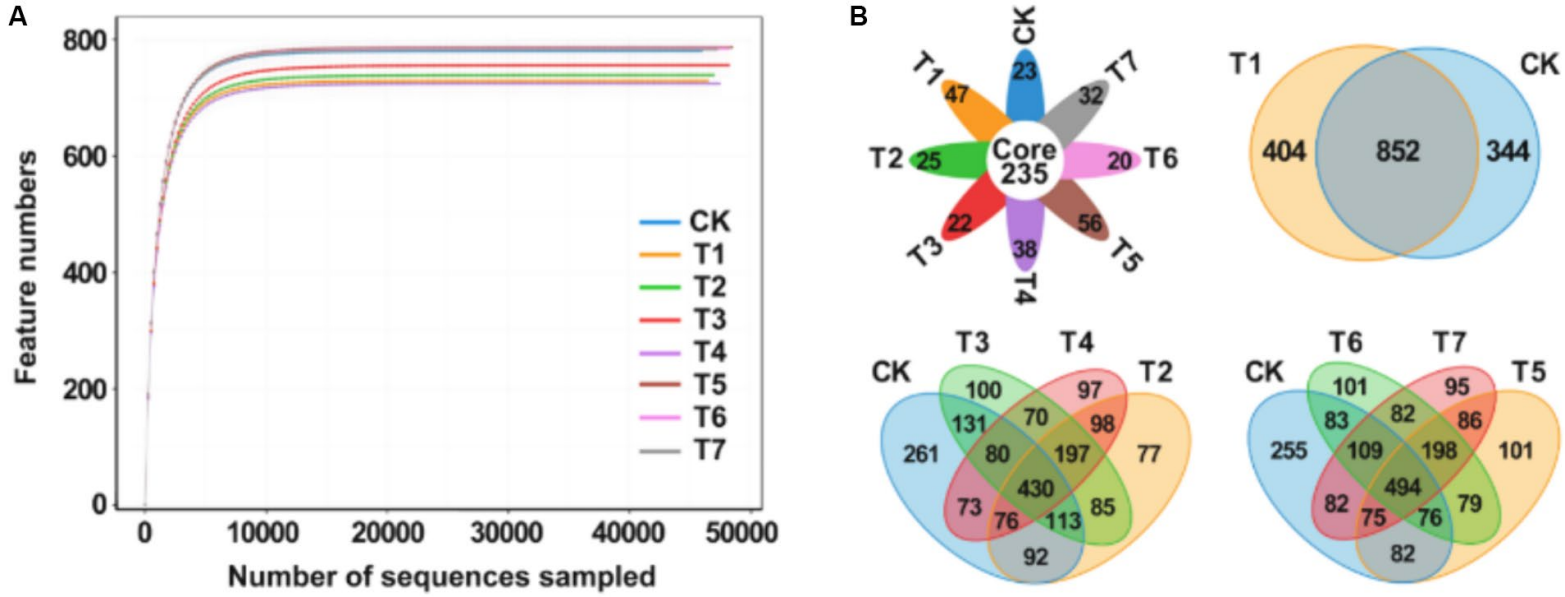
Analysis of rhizosphere soil bacterial community diversity under different fertilization treatment combinations. **(A)** Multi groups rarefaction curves of rhizosphere soil bacterial. **(B)** Venn analysis of soil bacterial community. T4, Half-supplied inorganic fertilizer + natural soil biotin; T7, half-supplied organic fertilizer+ natural soil biotin; T3, inorganic fertilizer + natural soil biotin; T2, inorganic fertilizer; T1, natural soil biotin; CK, no fertilization; T6, organic fertilizer + natural soil biotin; T5, organic fertilizer.

After high-throughput sequencing, a total of 1,921,600 gene sequences were obtained from the 8 sample groups. Following double-ended Reads quality control and splicing, 1,916,441 high-quality sequences were generated, with each sample sequence predominantly ranging between 400 bp and 450 bp in length. The dilution curve, illustrating the rate of new feature appearance with continuous sampling, is depicted in Figure 2A. As sequencing sequences increase, the curve tends to flatten, indicating that species richness in this environment does not continue to rise with increased sequencing quantity. These results suggest that the sample sequences are adequate for subsequent analysis. A Venn plot was employed to analyze differences in the number of bacterial amplification sequence variants (ASVs) in the rhizosphere soil of *Rhododendron* under different fertilization treatments (Figure 2B). The analysis revealed a total of 2,458 ASVs across the 8 samples, with 235 ASVs being characteristic, accounting for 9.6% of the total ASVs and ranging from 18.7 to 21.0% in each treatment group. Compared to the control group, the number of ASVs decreased in the fertilization treatment groups, with the T1 treatment exhibiting significantly fewer characteristic ASVs than CK. Additionally, the T2 treatment showed a significantly lower ASV count than CK. Following the application of inorganic fertilizers, the ASV count increased in T3, while it decreased in T4. A similar trend was observed in comparisons between the CK, T5, T6, and T7 groups.

The Alpha diversity index was employed to assess the species richness and diversity of soil bacterial communities across various fertilization treatments (Table 3). The soil bacterial community richness, as indicated by the Ace and Chao1 indices, spanned 725.33 to 787.71 and 725.33 to 787.67, respectively, with the sequence T5 > T6 > T7 > CK > T3 > T2 > T1 > T4. The application of inorganic fertilizer (T2, T3, T4) and natural soil biotin (T1) decreased bacterial richness, while organic fertilizer (T5, T6, T7) led to an increase. The T3 slightly enhanced richness over the T2, but the T4 saw a lower richness. Organic fertilizer treatments, including T5, T6, and T7, mirrored these findings. Diversity indices, Simpson ranging from 0.9966 to 0.9969 and Shannon from 8.884 to 9.066, followed the pattern T5 > T7 > CK > T6 > T1 > T2 > T3 > T4. The PD_whole_tree index, reflecting community diversity, descended through the treatments in the order of CK, T7, T6, T5, T1, T2, T3, to T4, with all but T7 showing a dip in diversity relative to CK.

**Table 3:** Alpha diversity index of soil bacterial community.

Treatment	Richness index		Diversity index		
	Ace	Chao1	Simpson	Shannon	PD_whole_tree
CK	781.75 ± 17.28^ab^	781.67 ± 17.21^ab^	0.9968 ± 0.0000^ab^	9.035 ± 0.031^a^	47.17 ± 0.78^a^
T1	730.00 ± 14.18^c^	730.00 ± 14.18^c^	0.9967 ± 0.0001^bc^	8.919 ± 0.021^b^	45.23 ± 0.90^ab^
T2	740.04 ± 12.11^bc^	740.00 ± 12.12^bc^	0.9967 ± 0.0001^ac^	8.918 ± 0.018^b^	44.04 ± 1.39^bc^
T3	757.14 ± 18.93^b^	757.33 ± 19.01^b^	0.9966 ± 0.0001^c^	8.914 ± 0.029^b^	43.22 ± 1.91^c^
T4	725.33 ± 19.40^c^	725.33 ± 19.40^c^	0.9967 ± 0.0001^bc^	8.884 ± 0.019^b^	41.34 ± 0.44^c^
T5	787.71 ± 3.26^a^	787.67 ± 3.21^a^	0.9969 ± 0.0001^a^	9.066 ± 0.013^a^	45.71 ± 0.30^ab^
T6	785.44 ± 12.61^a^	785.67 ± 12.66^a^	0.9966 ± 0.0002^c^	9.025 ± 0.046^a^	45.94 ± 1.12^ab^
T7	783.67 ± 12.90^a^	783.67 ± 12.90^a^	0.9967 ± 0.0001^abc^	9.042 ± 0.034^a^	46.39 ± 1.35^a^

T4, Half-supplied inorganic fertilizer + natural soil biotin; T7, half-supplied organic fertilizer + natural soil biotin; T3, inorganic fertilizer + natural soil biotin; T2, inorganic fertilizer; T1, natural soil biotin; CK, no fertilization; T6, organic fertilizer + natural soil biotin; T5, organic fertilizer. Different lowercase letters indicated a significant difference at 0.05 level.

**Figure 3:**
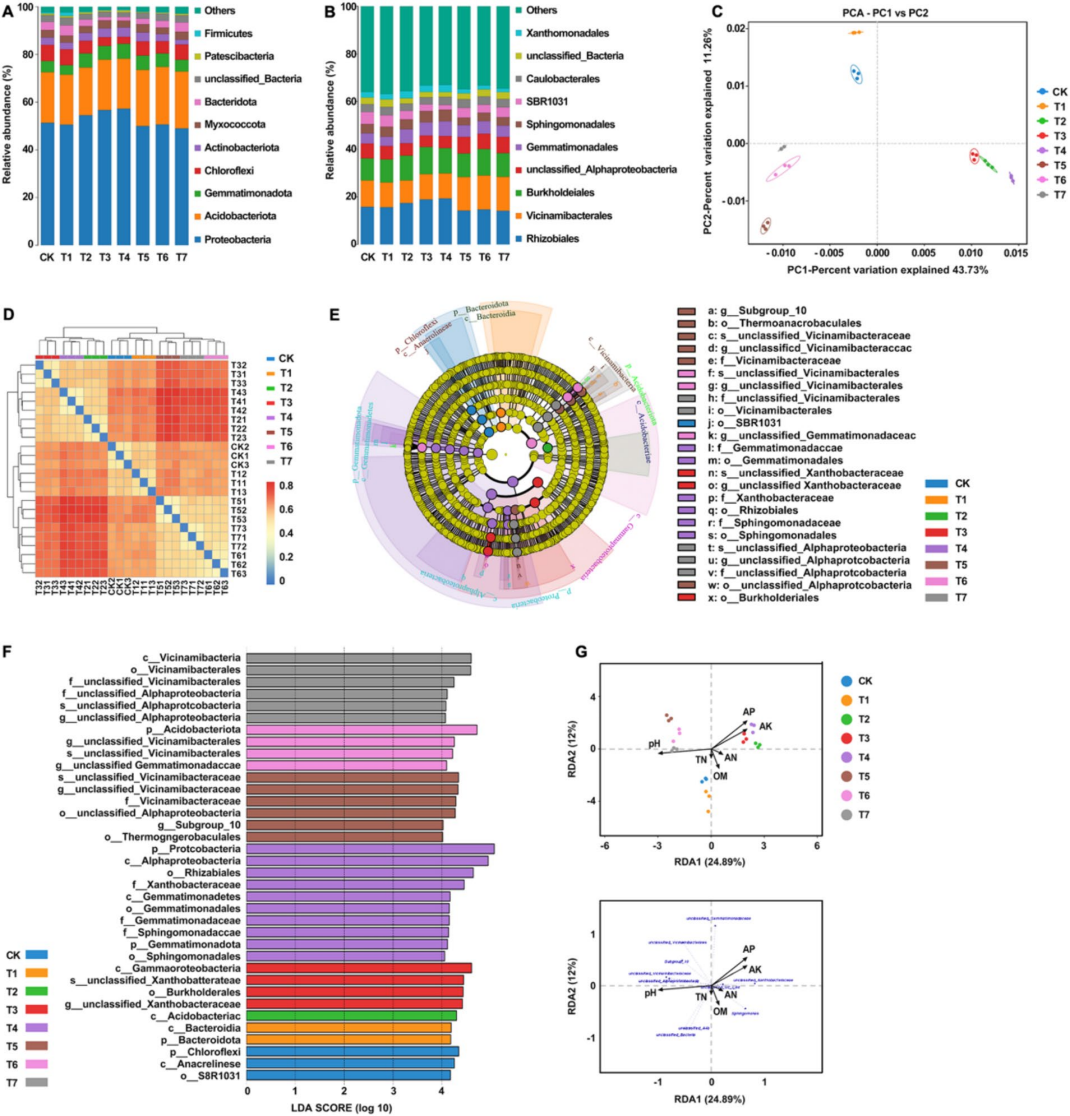
Analysis of the relationship between soil bacterial community structure and soil physical and chemical properties. **(A)** Relative abundances of the dominant bacterial phylum. **(B)** Relative abundances of the dominant bacterial order in soil. **(C)** PCA analysis between soil bacterial samples. **(D)** Heatmap of the soil samples. **(E)** Cladogram of soil bacterial community. The circles radiating from the inside out represent the classification level from phylum to species, and each small circle represents a classification at that level. Its diameter is proportional to relative abundance. Nodes of different colors represent the microbial communities that play an important role in the group represented by that color, while yellow represents no significant difference. **(F)** LDA value distribution histogram. **(G)** RDA analysis of bacterial communities and soil physicochemical properties at the genus level. T4, Half-supplied inorganic fertilizer + natural soil biotin; T7, half-supplied organic fertilizer+ natural soil biotin; T3, inorganic fertilizer + natural soil biotin; T2, inorganic fertilizer; T1, natural soil biotin; CK, no fertilization; T6, organic fertilizer + natural soil biotin; T5, organic fertilizer. The length of the arrow represents the impact of environmental factors, and the angle between the angle and the coordinate axis represents the correlation between the environmental factor and the coordinate axis. The smaller the angle, the higher the correlation. The closer the sample point is to the arrow, the stronger the effect of this environmental factor on the sample. The sample is located in the same direction as the arrow, indicating a positive correlation between environmental factors and changes in the sample species community, while the opposite is a negative correlation.

The feature sequences of each processed sample were classified and annotated, and the results showed that the species compositions of the treatment groups were similar but had differences in species abundance (Figure 3A). Among them, the dominant bacterial phyla in each treatment group included *Proteobacteria*, *Acidobacteria*, *Gemmatimonadota*, *Chloroflexi*, *Actinobacteriota*, *Myxococcota*, and *Bacteroidota*, accounting for 71.4–78.1% of the total relative abundance. Compared with CK, the relative abundance of *Proteobacteria* increased in the inorganic fertilizer treatment group (T2) and the mixed inorganic and natural soil biotin treatment group (T3, T4). The relative abundance of *Acidobacteria* was found to be increased in the organic fertilizer treatment group (T5, T6, and T7), and the results of the bacterial and inorganic fertilizer treatment group (T1, T2, T3, and T4) were similar to those of CK. In addition, the relative abundance of *Bacillus subtilis* and *Pseudomonas aeruginosa* decreased in the treatment groups, except for the natural soil biotin treatment group (T1), and the relative abundance of *Actinobacteria* increased in the treatment groups.

The bacterial orders with higher relative abundance in soil samples from each treatment group comprised *Rhizobiales*, *Vicinamibacterales*, *Burkholdeiales*, *Gemmatimonales*, *Sphingomonadales*, *SBR1031*, *Caulobacterales*, and *Xanthomonadales* (Figure 3B). Among them, the *Rhizobiales* and *Vicinamibacterales* order was the dominant bacterial order in various treatment groups (Supplementary Table 2). Beta diversity analysis was used to compare the similarity of species diversity among different samples, while Principal Component Analysis (PCA) was used to assess the differences between each group of data on a two-dimensional coordinate map. PC1 and PC2 axes were employed to capture the two feature values reflecting the maximum variance, with distinct treatments represented by different colors. Closer distances between data points indicated higher similarity among processed samples. As depicted in Figure 3C, the 8 sample groups primarily clustered into 3 distinct groups. The bacterial community structures of CK and T1 exhibited notable similarity, differing significantly from other treatments. T2, T3, and T4 demonstrated high similarity, while T5, T6, and T7 exhibited similar patterns. The heatmap, constructed based on a distance algorithm, facilitated the generation of a distance matrix between samples, allowing for an intuitive visualization of differences between two samples through changes in color gradients. Figure 3D illustrates that the results obtained from this approach for analyzing the similarity of bacterial community structures under various fertilization treatments were consistent with the PCA analysis. The Line Discriminant Analysis Effect Size (LEfSe) method was used to compare the bacterial community compositions of each treatment group, and the results showed significant differences (*p* < 0.05) among a total of 36 taxa under the set criteria (LDA > 4; Figures 3E,F). Redundancy analysis (RDA) was performed on the species diversity at the genus level among the samples to determine the correlation between bacterial communities and soil physicochemical properties (Figure 3G). The results showed that pH (*p* = 0.001), available P (*p* = 0.001), and available K (*p* = 0.001) were the main environmental factors affecting the structure of soil bacterial communities. The content of organic matter, total N, and hydrolytic N exhibited a positive correlation with the relative abundance of *Xanthomonadales*, unclassified A4b, unclassified Bacteria, and other bacterial genera. Conversely, the content of available P and available K was positively correlated with the relative abundance of unclassified *Xanthobacteriaceae* and unclassified *Gemmatimonadaceae* but negatively correlated with the relative abundance of unclassified A4b and unclassified Bacteria. The pH value was positively correlated with the relative abundance of unclassified *Alphaproteobacteria*, unclassified *Vicinamidobacteriaceae*, Subgroup_10, and other bacterial genera and negatively correlated with the relative abundance of unclassified *Xanthobacteriaceae*, unclassified SCI_84, and other bacterial genera. These findings suggest that different fertilization combinations exert an influence on the diversity of soil bacterial communities, with the application of natural soil biotin effectively reducing bacterial diversity in the rhizosphere soil. Specifically, the application of inorganic fertilizer alone or in combination with natural soil biotin increased the relative abundance of *Proteobacteria*, while the application of organic fertilizer increased the relative abundance of *Acidobacteria*. The dominant bacterial orders in the soil samples were *Bacteroidota*, *Gemmatimonales*, *Sphingomonadales*, *SBR1031*, *Caulobacterales*, and *Xanthomonadales*.

### 3.3 Effect of different fertilization treatment combinations on the fungal community in the rhizosphere soil of *Rhododendron*

**Figure 4:**
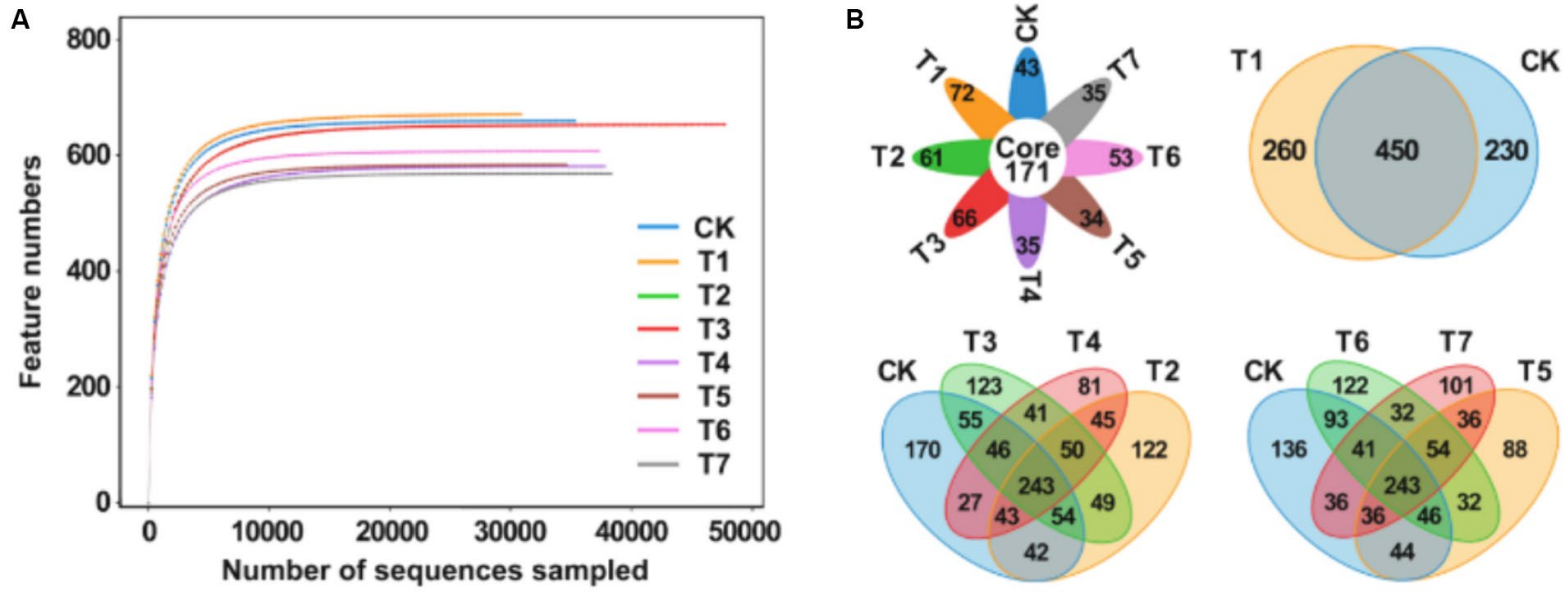
Analysis of rhizosphere soil fungal community diversity under different fertilization treatment combinations. **(A)** Multi groups rarefaction curves of rhizosphere soil fungal. **(B)** Venn analysis of soil fungal community. T4, Half-supplied inorganic fertilizer + natural soil biotin; T7, half-supplied organic fertilizer+ natural soil biotin; T3, inorganic fertilizer + natural soil biotin; T2, inorganic fertilizer; T1, natural soil biotin; CK, no fertilization; T6, organic fertilizer + natural soil biotin; T5, organic fertilizer.

A total of 1,349,294 high-quality sequences were generated from 8 sets of samples following double-ended Reads quality control and splicing. At the 97% similarity level, the dilution curves of each treatment flattened as sequencing numbers increased, indicating ample sequencing depth to reliably capture the results (Figure 4A). From the 8 groups of samples, 1,454 Amplicon Sequence Variants (ASVs) were identified. Specifically, the ASV counts for CK, T1, T2, T3, T4, T5, T6, and T7 treatments were 680, 710, 648, 661, 576, 579, 668, and 579, respectively, constituting 46.8, 48.8, 44.6, 45.5, 39.6, 39.8, 45.9, and 39.8% of the total ASVs (Figure 4B). Among these, 171 ASVs were shared across treatments, representing 11.8% of the total ASV count and 24.1–29.5% of the ASVs in each group. Notably, ASV counts decreased in treatments T1, T2, T3, and T4 compared to CK, except for a significant increase in T1. Treatment T6 exhibited a higher ASV count than T5, while T4 had the same ASV numbers as T5.

As shown in Table 4, the Ace and Chao1 indices reflecting soil fungi diversity across the 8 treatment groups ranged from 331.33 to 391.37 and 331.33 to 391.33, respectively, displaying the following overall performance trend: T1 > CK > T3 > T2 > T6 > T5 > T4 > T7. Compared with CK, treatment with T1 exhibited an approximately 1.8% increase in fungal community richness, while other treatments showed varying degrees of decrease. Notably, the mixed application of natural soil biotin (T3) slightly increased microbial community richness compared to the only use of inorganic fertilizer (T2), whereas richness was relatively lower in the treatment combining natural soil biotin with a reduction in inorganic fertilizer (T4). Similar trends were observed for organic fertilizer treatments (T5, T6, and T7). Regarding the Simpson and Shannon indices, values ranged from 0.9660 to 0.9890 and from 6.611 to 7.453, respectively, with the overall performance trend as follows: T1 > CK > T6 > T2 > T5 > T7 > T3 > T4. The PD_whole_tree index ranged from 95.12 to 115.64, with the overall performance trend: T1 > CK > T2 > T6 > T5 > T3 > T4 > T7. Except for T1, all treatments exhibited a decrease in diversity compared to CK.

**Table 4:** Alpha diversity index of soil fungal community.

Treatment	Richness index		Diversity index		
	Ace	Chao1	Simpson	Shannon	PD_whole_tree
CK	384.38 ± 26.25^a^	384.33 ± 26.27^a^	0.9886 ± 0.0009^a^	7.398 ± 0.049^a^	109.94 ± 5.18^ab^
T1	391.37 ± 66.60^a^	391.33 ± 66.61^a^	0.9890 ± 0.0016^a^	7.453 ± 0.252^a^	115.64 ± 21.19^a^
T2	380.38 ± 24.34^a^	380.33 ± 24.42^a^	0.9849 ± 0.0005^b^	7.134 ± 0.074^bc^	107.68 ± 3.34^ab^
T3	380.77 ± 22.62^a^	380.83 ± 22.68^a^	0.9713 ± 0.0015^d^	6.889 ± 0.062^d^	100.91 ± 3.63^ab^
T4	338.72 ± 23.70^a^	338.66 ± 23.71^a^	0.9660 ± 0.0038^e^	6.611 ± 0.086^e^	98.86 ± 3.02^ab^
T5	340.38 ± 14.09^a^	340.33 ± 14.01^a^	0.9844 ± 0.0004b^c^	7.075 ± 0.039^bcd^	105.01 ± 1.67^ab^
T6	354.11 ± 57.35^a^	354.00 ± 57.29^a^	0.9865 ± 0.0016^ab^	7.241 ± 0.250^ab^	106.67 ± 15.54^ab^
T7	331.33 ± 6.43^a^	331.33 ± 6.43^a^	0.9816 ± 0.0009^c^	6.918 ± 0.013^cd^	95.12 ± 4.80^b^

T4, half-supplied inorganic fertilizer + natural soil biotin; T7, half-supplied organic fertilizer + natural soil biotin; T3, inorganic fertilizer + natural soil biotin; T2, inorganic fertilizer; T1, natural soil biotin; CK, no fertilization; T6, organic fertilizer + natural soil biotin; T5, organic fertilizer. Different lowercase letters indicated a significant difference at 0.05 level.

**Figure 5:**
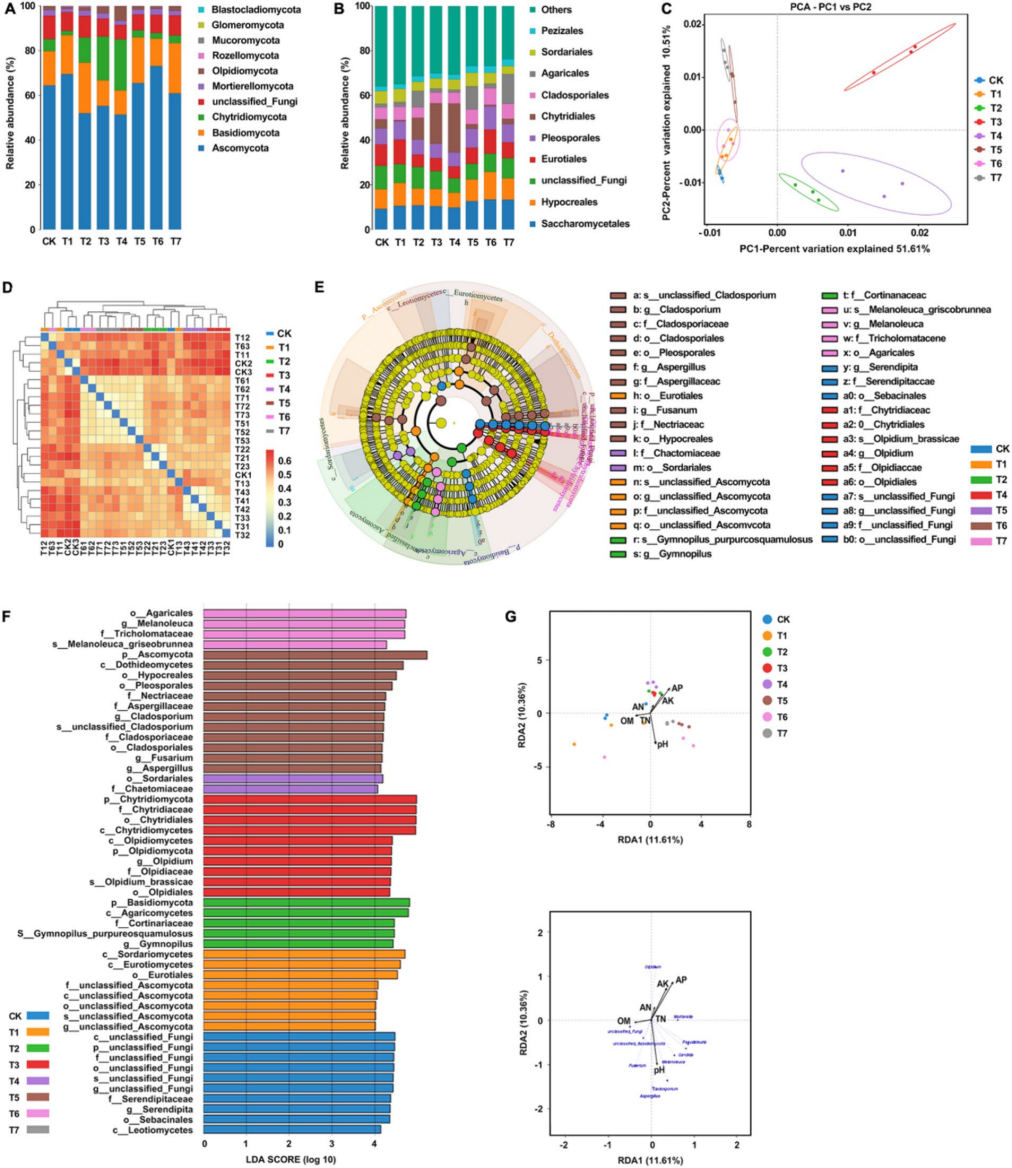
Analysis of the relationship between soil fungal community structure and soil physical and chemical properties. **(A)** Relative abundances of the dominant fungal phylum. **(B)** Relative abundances of the dominant fungal order in soil. **(C)** PCA analysis between soil fungal samples. **(D)** Heatmap of the soil samples. **(E)** Cladogram of soil fungal community. The circles radiating from the inside out represent the classification level from phylum to species, and each small circle represents a classification at that level. Its diameter is proportional to relative abundance. Nodes of different colors represent the microbial communities that play an important role in the group represented by that color, while yellow represents no significant difference. **(F)** LDA value distribution histogram. **(G)** RDA analysis of fungal communities and soil physicochemical properties at the genus level. T4, Half-supplied inorganic fertilizer + natural soil biotin; T7, half-supplied organic fertilizer+ natural soil biotin; T3, inorganic fertilizer + natural soil biotin; T2, inorganic fertilizer; T1, natural soil biotin; CK, no fertilization; T6, organic fertilizer + natural soil biotin; T5, organic fertilizer. The length of the arrow represents the impact of environmental factors, and the angle between the angle and the coordinate axis represents the correlation between the environmental factor and the coordinate axis. The smaller the angle, the higher the correlation. The closer the sample point is to the arrow, the stronger the effect of this environmental factor on the sample. The sample is located in the same direction as the arrow, indicating a positive correlation between environmental factors and changes in the sample species community, while the opposite is a negative correlation.

The taxonomic annotation results showed that the bacterial community composition structure of each treatment was similar, but the relative abundance of species varied greatly. The dominant bacterial phyla in each treatment included *Ascomycota*, *Basidiomycota*, *Chytridiomycota*, *Mortierellomycota*, and *Olpidiomycota*, with relative abundances higher than 10% (Figure 5A). The relative abundance of *Ascomycota* in each treatment group followed the order T6 > T1 > T5 > CK > T7 > T3 > T2 > T4. Notably, compared to CK, treatments involving inorganic fertilizers exhibited a significant decrease in relative abundance. Regarding *Basidiomycota*, the order of relative abundance was T2 > T7 > T5 > T1 > CK > T6 > T3 > T4, with the highest abundance observed when inorganic fertilizers were applied alone. For the phylum *Chlamydomonas*, the sequence of relative abundance was T4 > T3 > T2 > CK > T7 > T5 > T6 > T1, with a significant increase in abundance observed with inorganic fertilizer treatment, while a decrease was noted with organic fertilizer treatment. In the case of the *Aspergillus phylum*, the order of relative abundance was T6 > CK > T2 > T3 > T5 > T7 > T4 > T1, with a decrease observed in other treatments except for a slight increase in T6. Lastly, the relative abundance of the oil pot fungus phylum followed the order T4 > T3 > T7 > CK > T2 > T5 > T6 > T1, with T4 exhibiting notably higher abundance compared to other treatments. The dominant fungal orders in each treatment included *Saccharomycetes*, *Hypocreales*, *Eurotiales*, *Pleosporales*, *Chytridiales*, *Cladosporales*, *Agaricales*, *Sordariomycetidae*, and *Pezizomycetidae* (Figure 5B). Relative to CK, the abundance of yeast increased across all treatments, with a rise of 0.6 to 1.6% with inorganic fertilizer application, 3.4 to 4.2% with organic fertilizer, and 1.3% with natural soil biotin. Conversely, Streptomyces abundance decreased with inorganic fertilizer treatments but exhibited an increase in other treatment modalities. The abundance of *Pseudomonas aeruginosa* decreased in treatments with inorganic fertilizers but increased in those with organic fertilizers. Similarly, *Cladosporium* abundance decreased with inorganic fertilizer treatments but increased with organic fertilizer treatments. Treatments T5 and T7 exhibited the highest abundance of *Agaricales*, establishing them as the dominant fungi in these treatments. PCA analysis showed that the fungal community structures of CK, T1, and T6 were similar, while those of T2, T3, and T4 were significantly different (Figure 5C). Heatmap analysis corroborated these findings, indicating similarities between some samples from CK, T1, and T6 with those from other treatment groups, consistent with PCA results (Figure 5D). Figures 5E and 5F showed significant differences (*p* < 0.05) among a total of 51 taxa under the set criteria (LDA>4). RDA analysis showed that pH, available P, and available K were the main factors affecting the structure of soil fungal communities (Figure 5G). Organic matter was the main factor affecting the soil samples of CK and T1 while quick-acting P and K were the main factors affecting the soil samples of T2, T3, and T4. pH was the main factor affecting the soil samples of T5, T6, and T7. The organic matter content was positively correlated with the relative abundance of unclassified Fungi and unclassified *Basidiomycota*. The content of hydrolyzable N, available P, and available K were positively correlated with the relative abundance of *Olpidium* and negatively correlated with the relative abundance of *Fusarium* and unclassified *Basidiomycota*, and pH value was positively correlated with the relative abundance of *Aspergillus*, *Cladosporium*, *Candida,* and *Melanoluca*. These results indicate that the application of organic fertilizer and natural soil biotin can increase the number of ASVs and the diversity of the soil fungal community, while the application of inorganic fertilizer can reduce the number of ASVs and the diversity of the soil fungal community.

### 3.4 Effect of different fertilization treatment combinations on the growth of *Rhododendron*

The effects of different fertilization treatments on the growth of *Rhododendron* were examined over a 7-month cultivation period from April to October. This period, characterized by cool weather and suitable temperatures, coincides with the flowering phase of the Azalea. Following the flowering period, Azaleas began to produce new branches and foliage, resulting in rapid increases in plant height and width. Subsequently, during summer, the onset of continuous high temperatures and intense sunlight induces a dormancy phase in *Rhododendrons*, leading to a significant slowdown in growth rate. As temperatures cool down, typically in autumn, the growth rate of *Rhododendron* plant height and width accelerates once again. Eventually, with the arrival of winter, *Rhododendrons* enter another dormant period. Therefore, various growth indicators of *Rhododendron* were assessed during this period.

**Figure 6:**
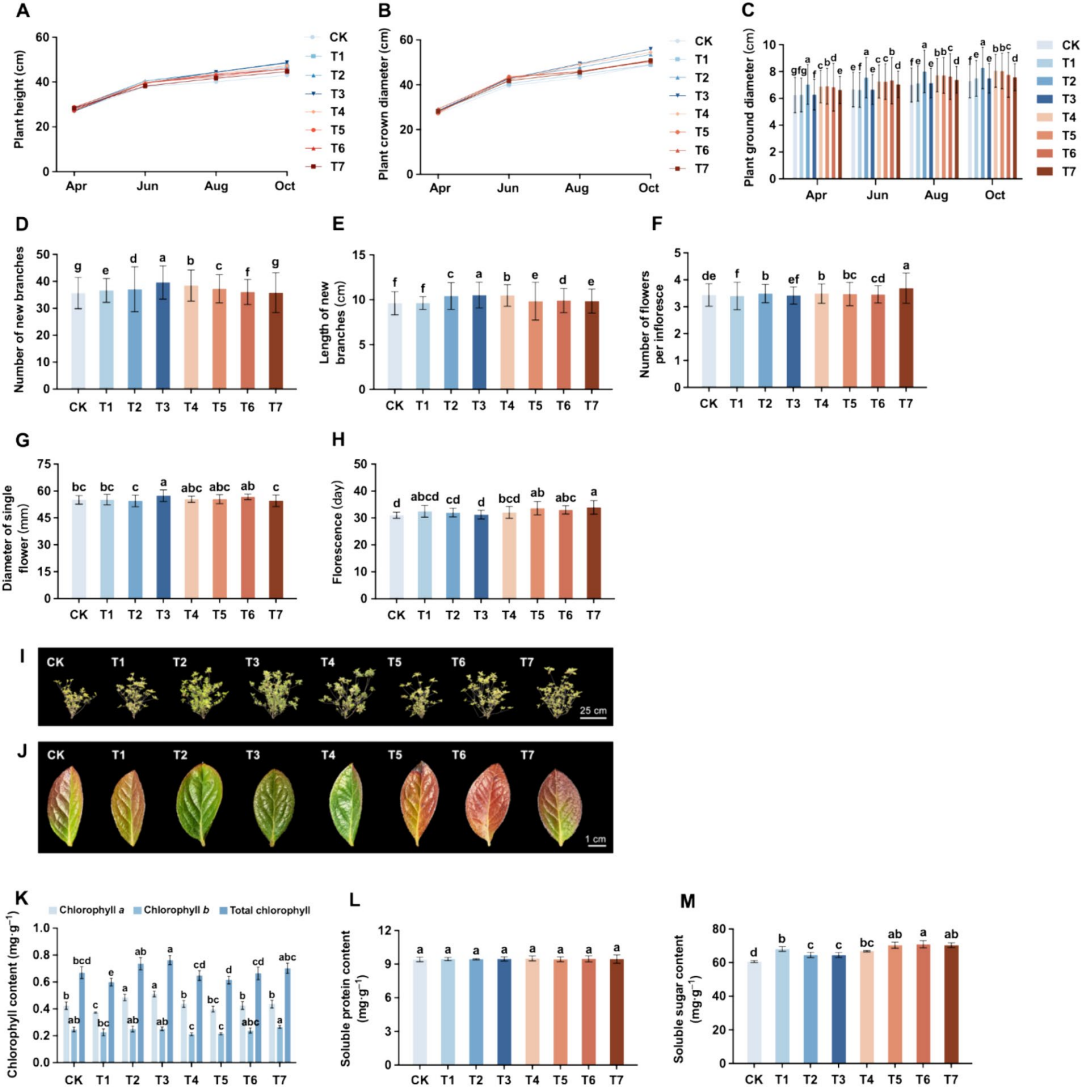
Effect of different fertilization treatment combinations on the growth of *Rhododendron*. **(A)** Plant height. **(B)** Plant crown diameter. **(C)** Plant ground diameter. **(D)** Number of new branches. **(E)** Length of new branches. **(F)** Number of flowers per inflorescence. **(G)** Diameter of a single flower. **(H)** Florescence. **(I)** Phenotype of *Rhododendron* cuttings after one-year trial. **(J)** Leaf color characteristic phenotype. **(K)** Chlorophyll content. **(L)** Soluble protein content. **(M)** Soluble sugar content. Different lowercase letters indicate significant differences between treatments in the same column (*p* < 0.05). T4, Half-supplied inorganic fertilizer + natural soil biotin; T7, half-supplied organic fertilizer+ natural soil biotin; T3, inorganic fertilizer + natural soil biotin; T2, inorganic fertilizer; T1, natural soil biotin; CK, no fertilization; T6, organic fertilizer + natural soil biotin; T5, organic fertilizer.

The plant height and width of *Rhododendron* in each treatment group are shown in Figures 6A,B. The plant height followed the order of T3 > T2 > T4 > T1 > T5 > T6 > T7 > CK, with a variation ranging between 43.07 cm and 48.71 cm. Compared with CK, the application of inorganic fertilizers (T2, T3, and T4) significantly increased plant height, while other treatments showed improvements, though the difference was not significant. Among them, the application of natural soil biotin alone (T1) increased plant height by 8.1%, while the application of organic fertilizers (T5, T6, and T7) increased plant height by 3.8 to 6.7%. The height of *Rhododendron* plants treated with mixed application of inorganic fertilizer and natural soil biotin (T3) was the highest, increasing by 12.5% compared to CK. The plant width followed the order of T3 > T4 > T2 > T5 > T7 > T6 > T1 > CK, with the variations ranging between 48.84 cm to 55.98 cm. Compared with CK, the application of inorganic fertilizers (T2, T3, T4) significantly increased the plant width, while other treatments showed no significant differences. Among them, the single application of natural soil biotin (T1) had the smallest increase of 0.3% in plant width, while the application of organic fertilizers (T5, T6, and T7) increased the plant width by 3.1 to 4.3%. The *Rhododendron* treated with mixed application of inorganic fertilizer and natural soil biotin (T3) had the highest plant width, an increase of 14.6% compared to CK. As shown in Figures 6C–6E, the number of new branches showed T3 > T4 > T5 > T2 > T1 > T6 > T7 > CK, while the length of new branches showed T3 > T4 > T2 > T6 > T7 > T5 > T1 > CK. Among all treatments, the mixed application of inorganic fertilizer and natural soil biotin (T3) had the greatest impact on the growth of new branches.

The effects of different fertilization treatments on the flowering of *Rhododendron* are shown in Figures 6F–6H, with the order being T7 > T4 > T2 > T5 > T6 > CK > T3 > T1. Concerning the size of individual flowers, the order was T3 > T6 > T5 > T4 > T1 > CK > T7 > T2. Notably, the treatment involving the mixed application of inorganic fertilizer and natural soil biotin (T3) exhibited the largest single flower diameter, significantly increasing by 4.2 and 5.2% compared to CK and the only application of inorganic fertilizer (T2), respectively. The results of other fertilization treatments did not significantly differ from CK. In terms of the flowering period duration, *Rhododendron* displayed the order T7 > T5 > T6 > T1 > T4 > T2 > T3 > CK, with all fertilization treatments extending the flowering period. Compared to CK, the application of inorganic fertilizers (T2, T3, and T4) prolonged the flowering period by 0.8–3.5%, while the sole application of natural soil biotin (T1) increased it by 4.7%, and the application of organic fertilizers (T5, T6, and T7) significantly increased it by 6.5%–9.4%. The longest flowering period (33.92 days) was observed in the treatment involving reduced organic fertilizer and mixed application of natural soil biotin (T7).

The leaves of *Rhododendron* ‘Carniation’ turned reddish brown in winter. During the experiment, it was found that although each treatment led to a color change, the timing and leaf color varied significantly. As shown in Figure 6J, by the end of November, the leaves of all treatments began to change color, with CK showing the earliest color change and the inorganic fertilizer treatments (T2, T3, and T4) showing the latest color change. By the end of January, the peak of leaf color change was observed, with the leaves of each treatment primarily turning reddish-brown. However, CK, natural soil biotin treatment (T1), and organic fertilizer treatments (T5, T6, and T7) exhibited red leaf coloration, whereas a portion of the leaves within the inorganic fertilizer treatments (T2, T3, and T4) remained dark green. In mid-February, the leaves of *Rhododendron* began to turn green, with the inorganic fertilizer treatments (T2, T3, and T4) showing the earliest color change, followed by CK and natural soil biotin treatment (T1), and the organic fertilizer treatments (T5, T6, and T7) showed the most delayed change. At this point, the disparity in leaf color between treatments was most apparent. CK’s leaves turned green near the petiole and veins, with reddish-brown edges. The leaves of the natural soil biotin treatment (T1) were similar to those of CK and tended to be yellowish. The leaves of the inorganic fertilizer treatments (T2, T3, and T4) had mostly turned green, with only the edges showing withered yellow or red. The leaves of the organic fertilizer treatments (T5, T6, and T7) were still mainly red in color. The leaves of the single application of organic fertilizer (T5) were yellowish-red with withered leaf tips. The leaves of the mixed application of organic fertilizer and natural soil biotin (T6) were uniformly orange-red in color. The leaves of the reduced application of organic fertilizer and natural soil biotin (T7) had turned green near the petiole, while the leaf tips and edges remained reddish brown. By the end of March, most of the leaves of *Rhododendron* in each treatment group had turned green. The effects of different fertilization treatments on the chlorophyll content of *Rhododendron* leaves are presented in Figure 6K. The changing trends of chlorophyll a, chlorophyll b, and total chlorophyll content were generally consistent. The total chlorophyll content ranking for each treatment is as follows: T3 > T2 > T7 > CK > T6 > T4 > T5 > T1. Notably, the mixed application of inorganic fertilizer and natural soil biotin (T3) exhibits the highest chlorophyll content, reaching 0.763 mg·g^−1^, representing a significant increase of 13.9% compared to CK. Conversely, the chlorophyll content of the single application of natural soil biotin (T1) was significantly decreased by 10.6% compared to CK, while the total chlorophyll content of other treatments showed no significant deviation from CK. Regarding soluble protein content, the order is as follows: T4 > T6 > T3 > T7 > T1 > T2 > T5 > CK (Figure 6L). Although there’s an overall improvement in soluble protein content compared to CK across all fertilization treatments, the differences are not statistically significant. Specifically, the soluble protein content of the single application of natural soil biotin (T1) increases by 0.3% compared to CK (9.42 mg·g^−1^). Compared with a single application of inorganic fertilizer (T2) and organic fertilizer (T5), the soluble protein content increased with the mixed application of natural soil biotin (T3, T6) or the reduced application of respective fertilizers combined with natural soil biotin (T4, T7). Notably, the soluble sugar content in the experimental groups surpassed that of the CK, with the order being T6 > T7 > T6 > T1 > T4 > T2 > T3 > CK (Figure 6M). The soluble sugar content of a single application of natural soil biotin (T1), inorganic fertilizer (T2), and organic fertilizer (T5) increased by 12.3, 6.5, and 16.0% compared to CK (60.64 mg·g^−1^), respectively. The highest soluble sugar content was found in using the T6 treatment with mixed application of organic fertilizer and natural soil biotin, which was 70.95 mg·g^−1^, a significant increase of 17.0% compared to CK. The soluble sugar content of inorganic fertilizer treatments (T2, T3, and T4) was lower compared to other fertilization treatments. The soluble sugar content of inorganic fertilizer treatments (T2, T3, and T4) was lower compared to other fertilization treatments. Compared with T2 (64.56 mg·g^−1^) using a single application of inorganic fertilizer, treatment T4, involving the reduction of inorganic fertilizer and mixed application of natural soil biotin, exhibited a significant increase in soluble sugar content by 3.5%.

It was found that the mixed application of inorganic and natural soil biotin significantly promoted the growth and flowering of *Rhododendron*, which could increase the height, width, number, and length of new branches, as well as the number and size of flowers. At the same time, reducing the amount of organic fertilizer and mixing it with natural soil biotin could also prolong the flowering period of *Rhododendron*. Under different fertilization treatments, the color change process of *Rhododendron* leaves was found to vary, and there are also differences in chlorophyll content, soluble protein content, and soluble sugar content. Among them, the mixed application of inorganic and natural soil biotin (T3) had a significant promoting effect on the nutrition and color of *Rhododendron* leaves.

## 4 Discussion

### 4.1 The application of natural soil biotin improved soil physicochemical properties and increased soil nutrient contents

As the foundation of agricultural ecosystems, soil directly impacts the growth and development of plants. Among them, the physical and chemical properties of soil play a crucial role in maintaining soil water and fertilizer capacity, its physical and chemical properties are pivotal in maintaining soil water and fertilizer capacity, nutrient supply, and regulating soil air and heat conditions (Delgado and Gómez, 2016; Kumar et al., 2022; Zhang X. et al., 2023). Fertilization is an important method for adjusting soil physical and chemical properties, but its effectiveness can significantly vary based on the type, amount, and timing of application. This experiment systematically investigated the impacts of diverse fertilization methods on soil pH and nutrient content through a series of fertilization treatments.

The pH value of soil is a key indicator for measuring soil acidity and alkalinity, directly influencing the availability of nutrients to plants. Optimal plant growth typically occurs within a pH range of 6 to 7, with deviations from this range potentially hindering growth (Zhang Y. et al., 2022). In this experiment, a substrate mixed with peat soil and pine scales was utilized for *Rhododendron* cultivation, considering the plant’s preference for acidic soil conditions. The initial pH value was 5.78 ± 0.01. By applying different fertilizers, it was observed that inorganic fertilizers notably decreased soil pH, potentially benefiting short-term *Rhododendron* growth but posing long-term risks such as soil acidification (Yao et al., 2011; Turner et al., 2020). Conversely, organic fertilizer application significantly raised soil pH, likely attributed to the production of humic acid during organic fertilizer decomposition, which enhances soil buffering capacity. Notably, the application of natural soil biotin alone had minimal impact on pH, suggesting its efficacy in preserving soil acid–base equilibrium.

In addition to pH value, soil nutrient content is also an important indicator for measuring soil fertility. In this experiment, all fertilization treatments were found to increase the nutrient content of the soil to a certain extent, but there were significant differences among different treatments. The application of natural soil biotin significantly increased soil organic matter and total N content, which was closely related to its function of promoting root absorption and plant growth. However, it is worth noting that the content of hydrolyzable N, available P, and available K did not increase as expected and sometimes even decreased, suggesting that when applying natural soil biotin, there is still a need to combine appropriate amounts of chemical fertilizer to ensure a balanced supply of nutrients (He, 2022). The inorganic fertilizer and organic fertilizer treatment groups showed different characteristics in improving soil nutrient content. Inorganic fertilizer had a particularly significant effect on improving available P and K levels, while the effects with organic fertilizer were relatively mild. When natural soil biotin is mixed with other fertilizers, the organic matter content and available nutrients in the soil are more abundant, which is more conducive to plant absorption and utilization.

Additionally, the focus was on soil enzymes, recognized as vital indicators of soil biological activity and integral to the myriads of biochemical reactions occurring within the soil. The vigor of these enzymes mirrors the soil’s biological activity and is crucial for the circulation of nutrients and energy conversion within the soil (Wang L. et al., 2023). Monitoring shifts in their activity provides valuable insights into the existing fertility levels and the ongoing changes within the soil. In this study, the investigation centered on changes in the activities of catalase, urease, and sucrase. The changes in catalase activity were closely related to soil pH value (Stpniewska et al., 2009), and the effects of different fertilization treatments on its activity showed significant differences. Urease is important for soil N cycling (Fang et al., 2022), and its activity can be influenced by various factors, including soil temperature, humidity, and water and fertilizer management measures. The change in sucrase activity reflects the transformation of organic carbon in the soil (Zhang Z. et al., 2022). The application of organic fertilizer significantly improved sucrase activity, possibly due to the increase in available carbon sources for microorganisms brought about by the addition of rich carbohydrates to the soil, thereby accelerating the rate of soil carbon cycling (Arnosti et al., 2021). In contrast, the application of inorganic fertilizer significantly decreased sucrase activity. However, the treatment that reduced the amount of inorganic fertilizer and mixed it with microbial fertilizer showed significantly higher sucrase enzyme activity than the treatment that applied inorganic fertilizer alone. This may be because urease activity is also influenced by soil temperature, humidity, and water and fertilizer management measures (Carlos et al., 2022; Kalala et al., 2022; Ni and Pacholski, 2022). Specifically, when the soil temperature is low and the amount of topdressing is large, the conversion rate of soil nitrate N may slow down, leading to accumulation and thus inhibiting urease activity. Soil enzyme activity is intricately connected to the levels and types of organic matter and N in the soil, serving as an indicator of the soil’s nutrient transformation efficiency.

Therefore, fertilization was observed to significantly influence soil physicochemical properties and enzyme activity. Through appropriate combinations of different fertilizers, soil quality and fertility can be effectively enhanced, thereby creating optimal conditions for plant growth. Specifically, the application of natural soil biotin, as a comprehensive soil management approach, not only balanced soil pH and increased nutrient content but also stimulated soil biological activity by modulating enzyme activity, thereby offering robust support for sustainable agricultural development. These findings provide an important basis for a deeper understanding of the impact of fertilization on soil and its relationship with plant growth, as well as theoretical guidance for the scientific and rational formulation of fertilization strategies.

### 4.2 The application of natural soil biotin reduced the relative abundance and diversity of soil bacterial communities while improving the relative abundance and diversity of fungal communities

As an ecosystem teeming with biodiversity, soil hosts a vast and diverse microbial community, which not only participates in the transformation and cycling of soil nutrients through metabolic activities but also actively promotes plant growth and development, making them of great significance for studying soil fertility status (Cheng et al., 2022; Chaudhary et al., 2023; Jiang et al., 2023). The rhizosphere, defined as the microenvironment surrounding the root system and heavily influenced by plant roots, serves as a dynamic interface facilitating material exchange between soil and plant ecosystems (Gregory, 2022). Root exudates supply ample carbon and N sources, fostering a microbial population within the rhizosphere that often surpasses that of non-rhizosphere microorganisms. Thus, distinct differences arise in the population structure and diversity of microorganisms inhabiting this zone. These rhizosphere microorganisms play important roles in modulating soil characteristics, thereby influencing plant physiological metabolism and indirectly shaping plant growth and development (Kong and Liu, 2022; Wang D. et al., 2022).

This study examined the impact of various fertilization methods on the bacterial and fungal community diversity within the rhizosphere soil of *Rhododendron*. The results revealed that the single application of natural soil biotin significantly reduced the relative abundance and diversity of soil bacteria while increasing the relative abundance and diversity of fungi. This effect could be attributed to the presence of specific functional microbial communities inherent in natural soil biotin. The application of natural soil biotin potentially stimulated the growth and metabolism of certain dominant microbial populations, consequently diminishing or even eliminating the presence of less advantaged microbial communities (Hao et al., 2023). In contrast, the application of inorganic fertilizer alone resulted in a significant decrease in the relative abundance and diversity of soil bacteria and fungi. This outcome may be attributed to the resultant decrease in soil pH following the application of inorganic fertilizer, which is unfavorable for the survival and proliferation of microorganisms (Lai et al., 2022). In the treatment using mixed application of natural soil biotin and reduced application of inorganic fertilizer, the decrease in soil bacterial and fungal relative abundance and diversity was reduced, indicating that the application of natural soil biotin to some extent alleviated the destructive effect of inorganic fertilizer on soil microbial communities. On the other hand, the application of organic fertilizers resulted in divergent effects on soil microbial communities. While there was a slight increase in bacterial relative abundance and diversity, there was a decrease in fungal relative abundance and diversity. Such variations may stem from the distinct impacts of organic fertilizers on the activity levels of different microbial communities (Holík et al., 2019).

Regarding bacterial community structure, different fertilization methods led to differences in the relative abundance of species in the rhizosphere soil of *Rhododendron*. Although the main components of soil microbial communities in each treatment were similar, the relative abundances of *Proteobacteria*, *Acidobacteria,* and *Actinobacteria* changed significantly. Among them, the application of inorganic fertilizer significantly increased the relative abundance of *Proteobacteria*, while the application of organic fertilizer reduced the relative abundance of *Acidobacteria*. N-fixing nutrient bacteria, including rhizobia within the *Proteobacteria phylum*, contribute to enhancing soil nutrient content and stimulating plant growth (Gopalakrishnan et al., 2015; Alhammad et al., 2023). The *Acidobacteria* phylum thrives in acidic conditions and may engage in ecological functions such as plant residue degradation and iron cycling (Ward et al., 2009; Wang et al., 2021). In terms of fungal community structure, the dominant phyla across all treatments are *Ascomycota* and *Basidiomycota*. *Ascomycota*, known for decomposing recalcitrant organic matter, plays a pivotal role in nutrient cycling (Beimforde et al., 2014; Rathnayaka et al., 2023). *Basidiomycota* contributes to the degradation of plant debris, thereby supporting ecological balance and material cycling (Manici et al., 2024a,b). In this experiment, the application of both natural soil biotin and organic fertilizer increased the relative abundance of *Ascomycota*. Additionally, the mixed application of natural soil biotin significantly increased the relative abundance of *Basidiomycota*, which is beneficial for organic matter decomposition and nutrient utilization.

In summary, the application of natural soil biotin in the rhizosphere soil of *Rhododendron* results in reduced richness and diversity of bacterial communities increased richness and diversity of fungal communities, and alterations in bacterial and fungal community structures. Notably, it enhances the relative abundances of *Proteobacteria*, *Ascomycota*, and *Basidiomycota*, which is beneficial for the decomposition of soil organic matter, promoting nutrient cycling, and supporting plant growth. Conversely, the application of inorganic fertilizers can detrimentally affect soil microbiota, whereas the combined use of natural soil biotin and reduced inorganic fertilizers can mitigate this damage. Organic fertilizers exhibit diverse effects on soil bacterial and fungal communities. Nonetheless, each treatment significantly influences the structure of the rhizosphere soil microbial community of *Rhododendron*, with these effects closely intertwined with changes in soil physicochemical properties.

### 4.3 Application of natural soil biotin promoted the growth and stress resistance of *Rhododendron*

In contrast to the emphasis on high yield and quality in field crops, flower cultivation prioritizes ornamental value, stress resistance, and simplified management practices (Darras, 2020). Horticultural researchers have long focused on enhancing flower vigor, prolonging the flowering period, and enhancing flower brightness. Fertilization, a pivotal agronomic practice, profoundly influences the growth and ornamental attributes of flowers (Kentelky and Szekely-Varga, 2021). Nevertheless, improper fertilization methods can result in stunted flower growth, diminished soil quality, and even odor issues.

This study systematically compared the effects of eight different fertilization treatments on the growth, development, and ornamental characteristics of *Rhododendron*. Parameters such as plant height, width, and diameter are crucial indicators reflecting the growth status of *Rhododendron* (Wang et al., 2014), collectively forming the plant type, which is pivotal in evaluating flower ornamental value (Ferrante et al., 2015). The experimental results showed that all fertilization treatments promoted *Rhododendron* plant height and widened plant width, with the mixed application of inorganic fertilizer and natural soil biotin yielding the most pronounced effect. However, under conditions of high temperature and aridity, the group treated with organic fertilizer was notably affected, likely due to heat release during the organic fertilizer decomposition process. Hence, caution is advised when utilizing organic fertilizers in such conditions. In addition to plant type, the number and length of new branches are also important parameters for evaluating the growth status of *Rhododendron* (Wang F. et al., 2022). In this experiment, all fertilization treatments increased the number and length of new branches to a certain extent, but the difference was not significant. This trend was consistent with the changes in plant height and width, further proving the promoting effects of reasonable fertilization on the growth of *Rhododendron*.

The ornamental value of flowers is not only reflected in their form but also closely related to the number, size, and flowering period of the flowers. In this experiment, the effects of each treatment on the number of flowers in a single inflorescence of *Rhododendron* were not significant, but the diameter and flowering period of a single flower were significantly correlated with the fertilization treatment. Notably, the mixed application of inorganic fertilizer and natural soil biotin significantly increased the diameter of a single flower and improved the ornamental appearance of *Rhododendron*. Moreover, the application of organic fertilizer substantially prolonged the flowering duration, with the treatment involving reduced organic fertilizer and mixed application of natural soil biotin exhibiting the longest flowering period, which effectively extends the ornamental duration of *Rhododendron*.

Additionally, the study assessed the effects of different fertilization treatments on the color of *Rhododendron* leaves. Chlorophyll, as a key pigment in photosynthesis, directly affects the photosynthetic efficiency and growth rate of plants (Zhang Z. et al., 2023). The study results indicated that the application of inorganic fertilizer and the combined application of natural soil biotin significantly increased the total chlorophyll content of *Rhododendron* leaves. In contrast, the sole application of natural soil biotin resulted in a decrease in chlorophyll content. This could be attributed to the close relationship between chlorophyll content and soil nutrient levels, with inorganic fertilizers being capable of rapidly enhancing soil fertility and stimulating chlorophyll synthesis (Alzamel et al., 2022). Although natural soil biotin can promote the absorption of nutrients by plants, it needs to be used in combination with inorganic or organic fertilizers; otherwise, it may lead to insufficient soil nutrients and affect the synthesis of chlorophyll. The control group and the treatment group subjected to bacterial and organic fertilizers exhibited notable leaf discoloration during winter, whereas the treatment group treated with inorganic fertilizers did not manifest this phenomenon, possibly due to the distinct effects of various fertilizers on plant physiological metabolism, though the precise mechanism warrants further investigation. The growth and development of plants are intricately linked to environmental conditions. When environmental changes surpass plants’ tolerance thresholds, stress ensues (El Rasafi et al., 2022), posing significant risks to plant health and potentially resulting in plant demise under severe circumstances. Low temperatures represent a primary adverse factor affecting plant growth, particularly amid the escalating instability of the global climate system. Anomalies in temperature and extreme weather events intermittently occur, posing considerable challenges to agricultural productivity. After exposure to cold stress, *Rhododendron*s may exhibit symptoms such as leaf shrinkage, browning, slowed growth, and diminished ornamental value, thereby severely constraining their widespread utilization in landscaping (Verleysen, 2005; Dixon, 2019).

The soluble protein and soluble sugar content of mature leaves from winter azaleas were analyzed to assess the impact of different fertilization treatments on azalea cold tolerance. Soluble proteins play an important role in maintaining plant cell osmotic regulation, preventing dehydration and freezing, and serving as vital physiological indicators of plant cold tolerance (Grgac et al., 2022). Previous studies have established a positive correlation between soluble protein content and plant cold tolerance under low temperatures (Zhang et al., 2015). In this study, while the soluble protein content in each fertilization treatment increased compared to the control group, the difference was not statistically significant. Soluble sugars serve as additional intracellular protective substances in plants under low temperatures. Cold conditions stimulate the hydrolysis of macromolecular organic compounds such as proteins and starch, thereby increasing the production of soluble sugars (Kaplan et al., 2006). Accumulation of soluble sugars can regulate cell osmotic potential, consequently enhancing plant cold resistance (Yooyongwech et al., 2009). In this experiment, the soluble sugar content significantly increased in each fertilization treatment. Notably, the treatment involving mixed organic fertilizer and natural soil biotin exhibited the highest soluble sugar content, with a significant increase of 17.0% compared to the control group. The increase in soluble sugar content in the organic fertilizer treatment exceeded that of the natural soil biotin treatment alone and surpassed that of the inorganic fertilizer treatment. This suggests that all fertilization treatments, to some extent, enhanced the cold tolerance of *Rhododendron*, with organic fertilizer exhibiting superior performance, while natural soil biotin and inorganic fertilizer performed slightly less effectively.

In summary, appropriate fertilization practices exerted a substantial influence on the growth, development, and ornamental attributes of *Rhododendron*. In practical cultivation, fertilization strategies should be strategized in accordance with soil conditions, climatic variations, and the specific growth requirements of *Rhododendrons* to optimize cultivation outcomes. Additionally, careful consideration must be given to fertilizer selection and application to prevent issues such as improper fertilization, which can result in plant damage and soil quality deterioration.

## 5 Conclusion

This study provides significant findings on the effects of diverse fertilization strategies on both *Rhododendron* growth and soil characteristics. The research meticulously evaluated the impact of single and combined applications of inorganic and organic fertilizers, alongside natural soil biotin, on the cultivation of *Rhododendron*, a popular ornamental species. Through comprehensive assessments of soil physicochemical properties, rhizosphere microbial dynamics, enzymatic activities, and key plant growth indicators, the study illuminated the intricate relationships between fertilization practices and plant health. Importantly, the study discovered that the application of natural soil biotin induced notable improvements in soil quality and significantly enhanced *Rhododendron* growth. The use of natural soil biotin was particularly effective in enriching the soil, promoting robust microbial communities, and increasing enzyme activities, which are indicative of soil fertility and plant vitality. The study also highlighted the potential of natural soil biotin as a sustainable alternative to traditional fertilizers, capable of reducing dependency on inorganic and organic fertilizers while maintaining or even improving plant growth metrics. The analysis of physiological parameters, including chlorophyll, soluble protein, and soluble sugar content, further underscored the positive influence of natural soil biotin on *Rhododendron*’s physiological status. These insights are crucial for the development of eco-friendly and efficient fertilization strategies tailored to ornamental plant cultivation. In conclusion, this study delivers valuable insights and practical recommendations for the horticultural industry. It advocates for the adoption of natural soil biotin as a means to bolster ornamental plant cultivation while ensuring environmental sustainability and economic viability. The findings lay a solid theoretical groundwork for the judicious use of fertilizers, enriching agricultural practices with a nature-oriented approach that prioritizes ecological harmony alongside productivity.

## Data availability statement

The original contributions presented in the study are included in the article/Supplementary material, further inquiries can be directed to the corresponding authors.

## Author contributions

ZT: Conceptualization, Data curation, Formal analysis, Funding acquisition, Investigation, Methodology, Project administration, Resources, Software, Supervision, Validation, Visualization, Writing – original draft, Writing – review & editing. LC: Conceptualization, Formal analysis, Methodology, Writing – original draft. SL: Data curation, Investigation, Methodology, Project administration, Writing – review & editing. KP: Conceptualization, Data curation, Formal analysis, Investigation, Methodology, Writing – original draft. DL: Conceptualization, Data curation, Formal analysis, Investigation, Methodology, Writing – original draft, Writing – review & editing. ZG: Funding acquisition, Resources, Writing – original draft. YW: Investigation, Methodology, Writing – original draft. LH: Conceptualization, Data curation, Formal analysis, Methodology, Resources, Writing – original draft. YC: Conceptualization, Funding acquisition, Investigation, Methodology, Project administration, Resources, Visualization, Writing – original draft, Writing – review & editing.

## Footnotes

^1^http://www.ncbi.nlm.nih.gov/sra/

^2^https://qiime2.org/
